# Development of a QuEChERS-Based UHPLC-MS/MS Method for Simultaneous Determination of Six *Alternaria* Toxins in Grapes

**DOI:** 10.3390/toxins11020087

**Published:** 2019-02-01

**Authors:** Wenbo Guo, Kai Fan, Dongxia Nie, Jiajia Meng, Qingwen Huang, Junhua Yang, Yuanyuan Shen, Emmanuel K. Tangni, Zhihui Zhao, Yongjiang Wu, Zheng Han

**Affiliations:** 1 Institute for Agro-food Standards and Testing Technology, Shanghai Key Laboratory of Protected Horticultural Technology, Shanghai Academy of Agricultural Sciences, Shanghai 201403, China; guowenbo@saas.sh.cn (W.G.); fankai1983@gmail.com (K.F.); niedongxia@saas.sh.cn (D.N.); mengjiajia@saas.sh.cn (J.M.); huangqingwen@zju.edu.cn (Q.H.); yangjunhua@saas.sh.cn (J.Y.); shenyuanyuan@saas.sh.cn (Y.S.); zhao9912@hotmail.com (Z.Z.);; 2 College of Pharmaceutical Sciences, Zhejiang University, Hangzhou 310058, China;; 3 Organic Contaminants and Additives, Sciensano, Tervuren 3080, Belgium; emmanuel.tangni@sciensano.be

**Keywords:** *Alternaria* toxins, grape, modified QuEChERS, UHPLC-MS/MS

## Abstract

A simple and reliable analytical method for the simultaneous determination of alternariol (AOH), altenuene (ALT), tentoxin (TEN), altenusin (ALS), tenuazonic acid (TeA), and alternariol monomethyl ether (AME) in grapes was developed by ultra-high-performance liquid chromatography–tandem mass spectrometry (UHPLC-MS/MS). A modified QuEChERS (quick, easy, cheap, effective, rugged, and safe) procedure with the extraction by acetonitrile and purification by sodium chloride (0.5 g) and anhydrous magnesium sulfate (0.5 g) was established to recover the six *Alternaria* toxins. After validation by determining the linearity (*R*^2^ > 0.99), recovery (77.8–101.6%), sensitivity (limit of detection in the range of 0.03–0.21 μg kg^−1^, and limit of quantification in the range of 0.09–0.48 μg kg^−1^), and precision (relative standard deviation (RSD) ≤ 12.9%), the analytical method was successfully applied to reveal the contamination state of *Alternaria* toxins in grapes. Among 56 grape samples, 40 (incidence of 71.4%) were contaminated with *Alternaria* toxins. TEN was the most frequently found mycotoxin (37.5%), with a concentration range of 0.10–1.64 μg kg^−1^, followed by TeA (28.6%) and AOH (26.8%). ALT (10.7%), AME (3.6%), and ALS (5.4%) were also detected in some samples. To the best of our knowledge, this is the first report about the *Alternaria* toxins contamination in grapes in China.

## 1. Introduction

*Alternaria* toxins, secondary metabolites produced by *Alternaria spp*, e.g., *A. alternata*, *A. tenuissima*, and *A. infectoria*—are frequently found in cereals, fruits, and vegetables [1,2]. The most important members include alternariol (AOH), alternariol monomethyl ether (AME), altenuene (ALT), altenusin (ALS), tentoxin (TEN), and tenuazonic acid (TeA) [3]. Acute and chronic ingestion of *Alternaria* toxins can be associated with carcinogenesis [4], teratogenesis [5,6], and cytotoxicity [7], as well as reproductive and developmental toxicities [8,9], and therefore pose high potential risks to human health. The European Food Safety Authority (EFSA) has established the threshold of toxicological concern (TTC) values as 2.5 ng kg^−1^ body weight per day for AOH and AME, and 1500 ng kg^−1^ for TeA [10]. Considering its widespread occurrence and intense toxicity, TeA, the most prevalent *Alternaria* toxin, has been registered as a toxic chemical by the Occupational Safety and Health Act (OSHA), and a maximal limit of 500 μg kg^−1^ was proposed by German federal state Bavaria in sorghum/millet-based infant food [11].

As a worldwide grown fruit, global grape production is over 3 million tons annually, of which 71% are used for wine-making, 27% for fresh consumption, and 2% for raisin producing [12]. Grapes and its derived products are susceptible to the infection of *Alternaria* spp during maturation, as well as post-harvest and during processing, when they are improperly stored. AOH and AME have been found in red and white wine with concentrations in the range of 0.03–19.4 ng mL^−1^ and 0.01–0.23 ng mL^−1^, respectively, and also in red and white grape juice samples with concentrations of 0.03–0.46 ng mL^−1^ and 0.01–39.5 ng mL^−1^, respectively [13]. TeA, AOH, and AME have also been found in raisins with the concentrations in the range of 6.9–594.4 μg kg^−1^, 0.3–13.5 μg kg^−1^ and 3.5–15.6 μg kg^−1^, respectively [14]. However, until now, the literature has been sparse on the presence of *Alternaria* toxins contaminating grapes, and it is ambiguous whether the risks come from the production process or from the original fruits. Hence, it is important to develop a reliable and accurate method for simultaneous determination of multiple *Alternaria* toxins, and to investigate their real contamination levels in grapes.

A variety of analytical methods, i.e., thin-layer chromatography (TLC) [15], gas chromatography [GC] [16], and high-performance liquid chromatography (HPLC) with different detectors [17,18], have been developed for determination of *Alternaria* toxins. The most frequently used technique for toxin separation is HPLC, as it combines high resolution with increasingly sophisticated automation. The availability of different ionization sources, i.e., electrospray (ESI) and atmospheric pressure chemical ionization (APCI), has drastically improved the possibilities of employing HPLC-tandem mass spectrometry (HPLC-MS/MS) in *Alternaria* toxin analysis, resulting in enhanced performance, providing additional selectivity, and generating information with a high degree of structural specificity. Hitherto, most of the previous reports only focused on TeA, AOH, and AME [13,17,19], and other important *Alternaria* toxins (e.g., TEN, ALS, and ALT) were not investigated. Recently, an UHPLC-MS/MS method was established for the detection of various *Alternaria* toxins in wine, vegetable juices, and fruit juices [20]. The low recovery of ALS and tedious sample pretreatment procedures made this method unsuitable for analysis of the targeted *Alternaria* toxins in grapes.

The major objective of this study is to develop a rapid and reliable ultra-high performance liquid chromatography tandem mass spectrometry (UHPLC-MS/MS) method for simultaneous determination of AOH, AME, ALT, ALS, TEN, and TeA based on a simple sample preparation of modified QuEChERS (quick, easy, cheap, effective, rugged and safe) approach, and to explore the actual contamination situations of *Alternaria* toxins in grapes for the first time in the world. 

## 2. Results and Discussion

### 2.1. Optimization of the Ultra-High-Performance Liquid Chromatography–Tandem Mass Spectrometry Conditions

MS/MS parameters were optimized by flow injection analysis of an individual *Alternaria* toxin standard at a concentration of 50–200 ng mL^−1^. The precursor ions and cone voltage were optimized by MS scan acquisition, both in positive and negative ionization modes. Then, MS/MS scan acquisitions were applied to find the optimum product ions and collision energies (CE), cone voltages, and dwell time (Figure S1). As shown in Table 1, the majority of *Alternaria* toxins displayed better specificity and selectivity in ESI^+^, except for ALS, which showed stronger signals and lower background interference in ESI^−^.

**Figure 1:**
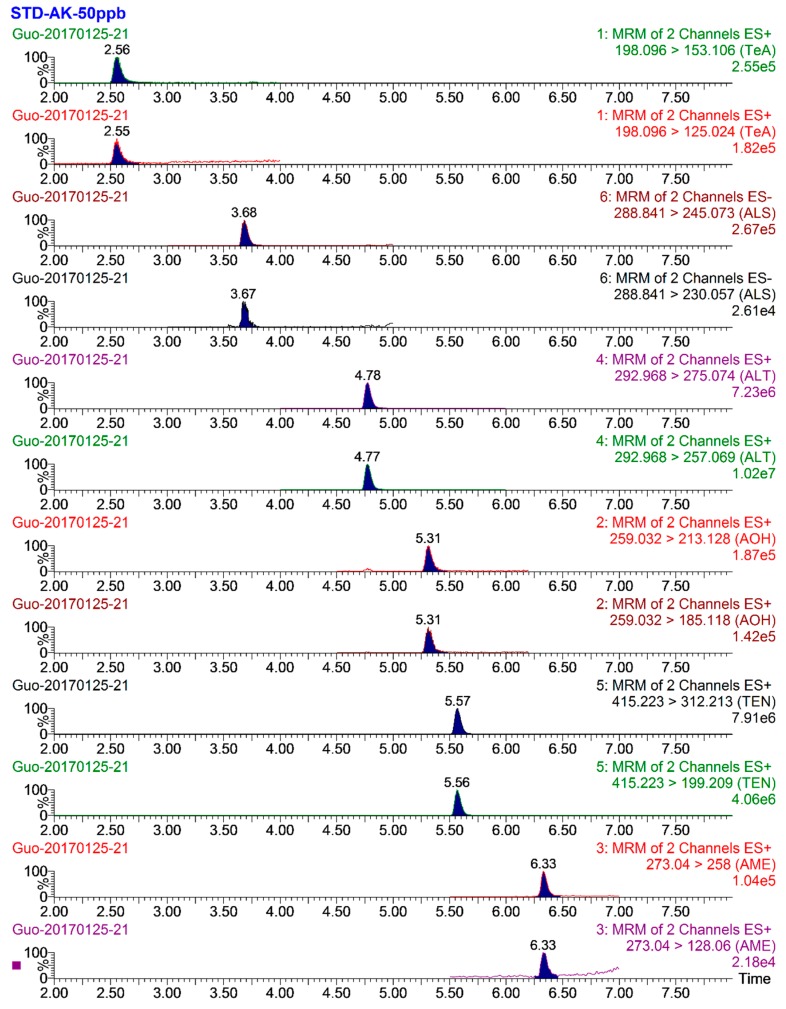
Multiple reaction monitoring (MRM) chromatograms of the six *Alternaria* toxins in the mixed standard solution. The concentration is 50 ng mL^−1^.

To obtain good chromatographic separation with symmetry and a sharp peak shape for targeted analytes, the compositions of the mobile phase and the chromatographic columns were optimized. Different additives—e.g., formic acid, ammonium formate, and ammonium acetate—were primarily evaluated. The highest ionization efficiency and sensitivity were obtained for all *Alternaria* toxins when methanol (A) and water containing 5 mmol L^−1^ aqueous ammonium acetate methanol (B) were used as the mobile phase. Subsequently, various chromatographic columns, i.e., the Waters ACOUITY UPLC BEH C_18_ column (100 mm × 2.1 mm, 1.7 μm), Agilent Proshell EC_18_ column (50 mm × 2.1 mm, 2.7 μm), and Waters ACQUITY UPLC^®^ HSS T3 (50 mm × 2.1 mm, 2.7 μm), which represented different stationary phases, were compared. Considering the efficient separation and response values, shown in Figure 1, the Proshell EC_18_ column (50 mm × 2.1 mm, 2.7 μm) was finally selected.

### 2.2. Optimization of the Sample Pretreatment Method

**Figure 2:**
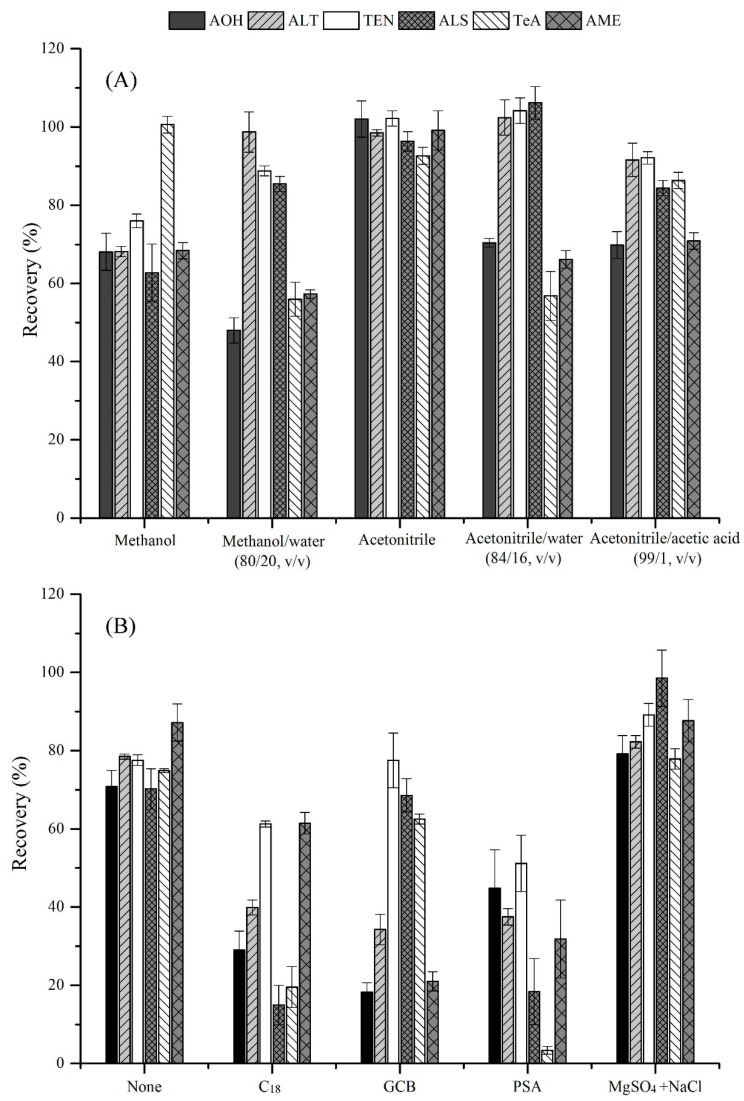
Comparison of the extraction efficiencies of different solvents (**A**) and purification efficiencies of different materials (**B**), using spiked grape samples. The concentration is 50 μg kg^−1^ (*n* = 6).

Establishment of an efficient sample pretreatment method is always the bottleneck for the development of an accurate and sensitive analytical method, due to the diversity of the physical and chemical properties of various *Alternaria* toxins, along with the complex sample matrices. In this study, five different frequently used extraction solvents, including methanol, methanol/water (80/20, *v*/*v*), acetonitrile, acetonitrile/water (84/16, *v*/*v*), and acetonitrile/acetic acid (99/1, *v*/*v*) were evaluated by using the blank grape samples spiked with 50 μg kg^−1^ of the targeted *Alternaria* toxins. As shown in Figure 2A, when methanol or methanol/water (80/20, *v*/*v*) were used, unsatisfactory recoveries (48.0–76.0%) were obtained for AOH, TeA, and AME. In addition, the extraction was emulsified with high contents of pigments and sugar. When acetonitrile was used as the extraction solvent, the recoveries were significantly increased (92.7–102.2%), especially for AOH and AME, against the recoveries of 70.4% and 66.2% for acetonitrile/water (84/16, *v*/*v*), and 69.8% and 70.9% for acetonitrile/acetic acid (99/1, *v*/*v*). As a consequence, acetonitrile was selected as the optimal extraction solvent.

**Figure 3:**
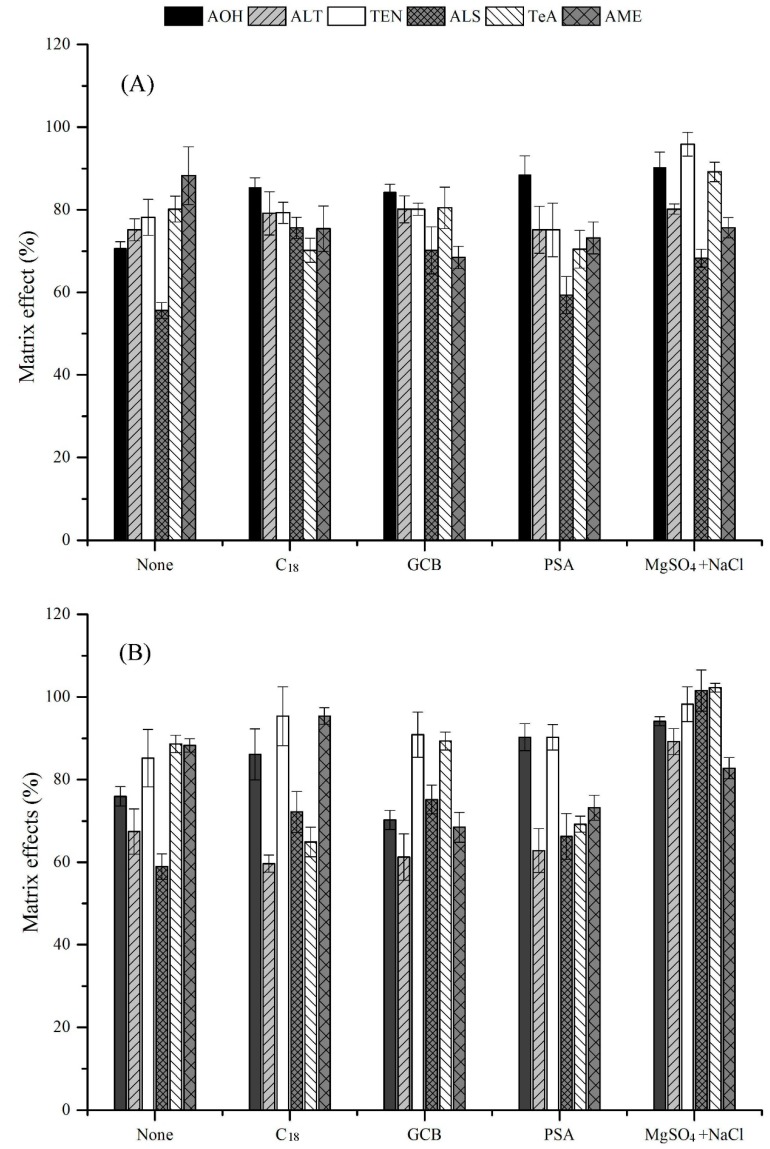
Matrix effects of the six *Alternaria* toxins in green grapes (**A**) and red grapes (**B**), purified by different materials (*n* = 6).

For sample clean-up, a modified QuEChERS method was developed to enrich the targeted analytes, and to remove the co-extractives as completely as possible. Different materials, including graphitized carbon black (GCB) (0.5 g), primary secondary amine (PSA) (0.5 g), C_18_ (0.5 g), MgSO_4_ (0.5 g), and NaCl (0.5 g) were tested for their purification efficiency. Unsatisfactory recoveries of 18.2–77.5% and 3.4–51.2% were obtained (Figure 2B) by using GCB and PSA, respectively, which had been used frequently to remove chlorophylls from fruits and vegetables in the previous studies [21]. The poor purification effects in the current work might be because of the π–π interactions through the sp^2^ hybrid orbitals of GCB and the planar aromatic compounds (i.e., AOH, AME, and ALT), as well as the ionic affinity between the amines in PSA and the carboxyl group in ALS [22,23,24]. With regard to C_18_, this material showed poor recoveries in the range of 15.0–61.5%. Finally, the salting-out step with anhydrous MgSO_4_ and NaCl was employed with the highest recoveries. in the range of 77.9–98.5% (Figure 2B), and lowest matrix effects, in the range of 82.8–102.3% (Figure 3).

Different membrane filters for filtering the re-dissolved solutions before injection into UHPLC-MS/MS, including nylon, poly tetra fluoroethylene (PTFE), mixed cellulose membrane (MCM), and polyvinylidene difluoride (PVDF), were compared. As shown in Figure S2, all membrane filters could be used for filtering ALT, TEN, ALS, and TeA. However, when nylon, MCM, and PVDF were applied, the recoveries were unsatisfactory for AOH and AME (3.6–19.0%). Satisfactory recoveries in the range of 86.1–100.5% for all *Alternaria* toxins were achieved when a PTFE membrane filter was selected.

### 2.3. Method Validation

The linearity of the six analytes in neat solvent and in matrix is shown in Table 2. Good linear relationships with correlation coefficients *R*^2^ > 0.99 were obtained. The limit of detection (LOD) and limit of quantification (LOQ) values were in the range of 0.03–0.21 μg kg^−1^ and 0.09–0.48 μg kg^−1^, respectively. The recoveries and precisions for the six *Alternaria* toxins at the three fortified levels are listed in Table 3. The mean recovery values ranged from 78.4% to 101.6% for green grapes, and 77.8% to 100.1% for red grapes. Intra- and inter-day precision was in the range of 2.5–12.2% and 3.7–12.9% for green grapes, and 1.9–11.4% and 2.9–10.8% for red grapes. Overall, the validation data indicated that the accuracy, repeatability, and sensitivity of the proposed method were acceptable and in agreement with the requirements of European Commission Decision 2002/657/EC (EC 2002). The current method could be used for the accurate detection of six *Alternaria* toxins in grapes.

### 2.4. Method Application

**Figure 4:**
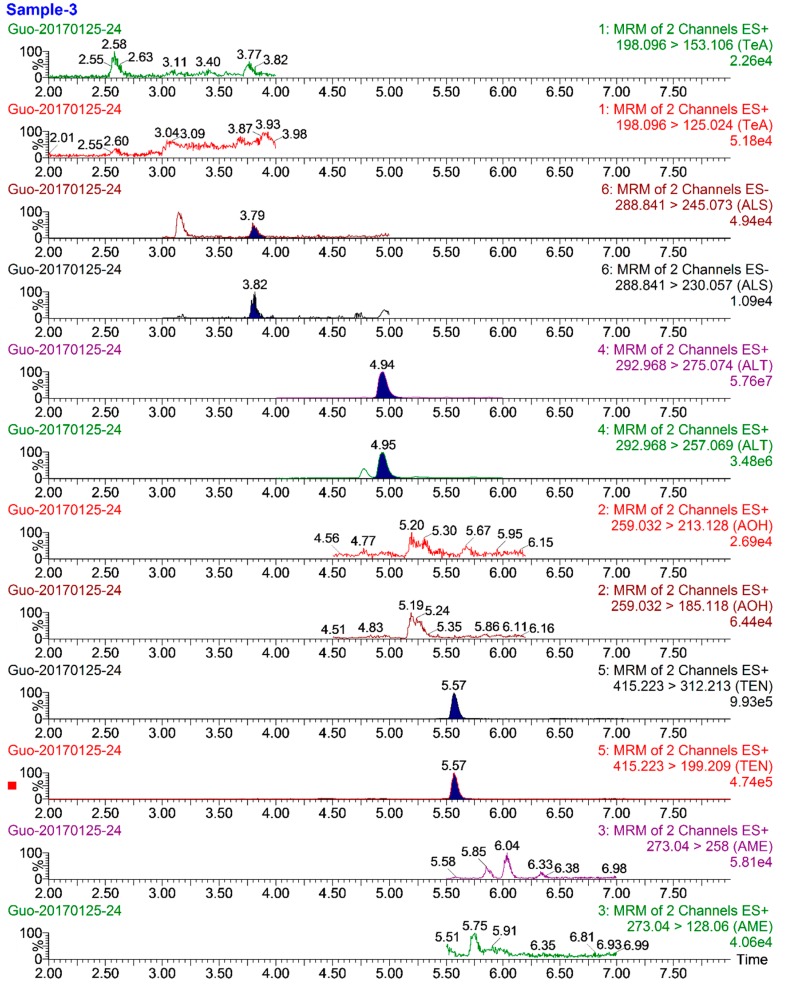
MRM chromatograms of a typical contaminated grape sample (No.3).

The validated method was applied to detect six *Alternaria* toxins in 56 grape samples randomly collected from the different markets and vineyards in Shanghai. MRM chromatograms of a typically contaminated grape sample are shown in Figure 4. The occurrences and concentration levels of the six *Alternaria* toxins are summarized in Table 4.

Among the 56 samples, 40 (incidence of 71.4%) were contaminated *Alternaria* toxins. TEN was the most frequently found mycotoxin (incidence of 37.5%), with concentrations in the range of 0.10–1.64 μg kg^−1^, followed by TeA (28.6%) and AOH (26.8%), with concentrations of 0.25–46.97 μg kg^−1^ and 0.09–7.15 μg kg^−1^, respectively. ALT (10.7%), AME (3.6%), and ALS (5.4%) were also detected in some samples. The contamination situations of *Alternaria* toxins revealed in the current work were in great agreement with the microbial status reported in the previous studies, in which, the toxigenic fungi, especially *Alternaria* spp., that could produce ALT, AOH, AME and TeA, were recovered from grapes [25,26]. It is not surprising to find so many *Alternaria* toxins in grapes, since large amounts of these toxins have been detected in grape juice and red/white wine with the incidences of almost 100% [13,17,19,20,27]. Compared to the contaminations in grape derivatives, the obviously lower incidences of AOH, TeA, and TEN in the original fruit were possibly because the production of *Alternaria* toxins occurred during the processing and storage processes. It is noteworthy that the grapes are frequently contaminated with multiple *Alternaria* toxins, and there is a need to improve prevention and control strategies during pre- and post-harvest procedures.

## 3. Conclusions

An accurate and reliable UHPLC-MS/MS method based on a modified QuEChERS technique was developed for the simultaneous determination of six *Alternaria* toxins in grapes for the first time. The method was proven to be simple, efficient, and accurate after validation by the determination of linearity, accuracy, and precision, and is feasible in practical grape samples. The survey results strongly suggested that the grape is a favorable matrix for *Alternaria spp* producing *Alternaria* toxins, and emphasizes the necessity of the current established method, which could be used for continuous monitoring of *Alternaria* toxins and reducing the health risk to consumers in China.

## 4. Materials and Methods

### 4.1. Chemicals and Reagents

The analytical standards (stock solutions) of AOH (100.0 μg mL^−1^), AME (100.3 μg mL^−1^), TEN (100.4 μg mL^−1^), and TeA (101.1 μg mL^−1^), dissolved in acetonitrile, were purchased from Romer labs (Union, MO, USA). Solid portions of ALT (99.4%) and ALS (98.0%) standards were purchased from AdipoGen (Liestal, Basel, Switzerland). The chemical structures of the six *Alternaria* toxins are shown in Figure S3.

Acetonitrile and methanol (HPLC grade) from Merck (Darmstadt, Germany) were used. Anhydrous magnesium sulfate (MgSO_4_, analytical grade), sodium chloride (NaCl, analytical grade), and ammonium acetate (HPLC grade) were supplied by ANPEL (Shanghai, China). Water used throughout the whole experiment was prepared by a Milli-Q system (Millipore, Billerica, MA, USA).

### 4.2. Preparation of Standard Solution

Solid portions of the ALT and ALS standards were dissolved in acetonitrile to prepare 100.0 μg mL^−1^ of stock solutions. A mixed standard solution of AOH, AME, ALT, ALS, TEN, and TeA with a concentration of 1 μg mL^−1^ was prepared in acetonitrile by diluting and mixing appropriate amounts of stock solutions of *Alternaria* toxins, and stored at −20 °C until use.

### 4.3. Samples Collection

A total of 56 grape samples, including 9 varieties (Kyoho, Summer Black, Shenhua, Hupei No.1, Shenfeng, Muscat Hamburg, Shenyu, Zuijinxiang, and Gold Finger) were randomly collected from different markets and vineyards in Shanghai. Approximately 0.5 kg of each sample was collected and mashed by a food processer (Midea, Guangdong, China). All samples were stored in a freezer at −20 °C until analysis.

### 4.4. Sample Preparation

The homogenized grape samples (2.0 g) were weighted into a 50 mL centrifuge tube and 10 mL of acetonitrile was added. The mixture was shaken at 200 rpm for 30 min. Subsequently, 0.5 g anhydrous magnesium sulfate and 0.5 g sodium chloride were added to the slurry and vigorously shaken for 30 s immediately. After centrifugation at 4500 rpm for 10 min, 5 mL of the supernatant was collected and evaporated under a soft stream of nitrogen gas at 40 °C. The residue was re-dissolved with 1 mL acetonitrile/water containing 5 mmol L^−1^ ammonium acetate (20/80 *v*/*v*), and filtered through a 0.22 μm PTFE membrane filter to be ready for analysis.

### 4.5. UHPLC–MS/MS Analysis

UHPLC analysis was performed on a Waters ACQUITY Ultra High-Performance LC system (Waters, Milford, MA, USA). Chromatographic separation was achieved on a Proshell EC_18_ column (50 mm × 2.1 mm, 2.7 μm). The mobile phase was consisted of methanol (A) and water containing 5 mmol L^−1^ ammonium acetate (B). A linear gradient elution program was set as follows: initial 10% A; 1 min, 10% A; 5 min, 90% A; 6 min, 90% A; 6.5 min, 10% A; 8 min, 10% A. The flow rate was 0.4 mL min^−1^. The injection volume was 3 μL, and the column temperature was 35 °C.

For MS/MS detection, a Waters T-QS mass spectrometer system (Waters, Milford, MA, USA) was used both in positive electrospray ionization mode (ESI^+^) and in negative electrospray ionization mode (ESI^−^) with the following parameters: interface voltages of capillary, 2.5 kV(ESI^+^) and 1.5 kV(ESI^−^); desolvation temperature, 500 °C; and source temperature, 150 °C. The gas flow rates were 7.0 bar for nebulizing gas and 1000 L h^−1^ for desolvation gas, respectively. Multiple reaction monitoring (MRM) mode was used for the quantification and confirmation of the *Alternaria* toxins with the parameters shown in Table 1.

### 4.6. Method Validation

The proposed method was validated by determination of the linearity, sensitivity, recovery, precision, and matrix effect according to the recommendations of European Commission Decision 2002/657/EC [28]. Different concentrations (0.1, 0.2, 0.3, 0.5, 1, 5, 10, 50, 100, and 200 ng mL^−1^) of *Alternaria* toxins were freshly prepared by diluting the working solution step by step with acetonitrile and a blank matrix, respectively. The calibration curves were constructed by plotting the responses versus analyte concentrations. The sensitivity was evaluated by determining the limit of detection (LOD) and limit of quantification (LOQ), which were designed as the concentrations of the toxins that produced signal-to-noise ratios (S/N) of 3 and 10 in matrix, respectively. The recoveries were tested using non-contaminated grape samples spiked with low, intermediate, and high concentration levels (10, 50, and 100 μg kg^−1^) of *Alternaria* toxins. The intra- and inter-day precisions were evaluated through the relative standard deviations (RSDs), using the non-contaminated samples spiked with different concentrations (10, 50, and 100 μg kg^−1^) of *Alternaria* toxins in the same day and in five successive days, respectively. All experiments were performed in sextuplicate.

Signal suppression and enhancement (SSE) was used to evaluate the matrix effect, which was calculated according to Equation (1) [21]:

SSE (%) = 100 × slope _matrix_ / slope _solvent_ (1)

where slope _matrix_ is the slope of matrix-matched calibration curve, and slope _solvent_ is the slope of standard calibration curve.

## Figures and Tables

**Table 1:** Tandem mass spectrometry (MS/MS) parameters for the determination of six *Alternaria* toxins.

*Alternaria* Toxins	Precursor Ions (*m/z*)	Product Ions (*m/z*)	Dwell Time (s)	Cone Voltage (V)	Collision Energy (eV)
AOH	259.0 [M+H]^+^	185.1 *	0.025	64	28
		213.1	0.025	64	24
AME	273.0 [M+H]^+^	128.1 *	0.030	54	26
		258.0	0.030	54	25
ALT	293.0 [M+H]^+^	257.1 *	0.025	4	14
		275.1	0.025	4	8
TEN	415.2 [M+H]^+^	199.2 *	0.029	32	12
		312.2	0.029	32	18
TeA	198.1 [M+H]^+^	125.0 *	0.025	42	16
		153.1	0.025	42	12
ALS	288.8 [M−H]^−^	235.1 *	0.029	−8	−20
		245.1	0.029	−8	−16

* Primary product ion.

**Table 2:** Linearity, limit of detection (LOD) and limit of quantification (LOQ) of different *Alternaria* toxins.

*Alternaria* Toxins	Neat Solvent				Green Grape				Red Grape			
	Linear Range (ng mL^−1^)	*Correlation Coefficient* (*R*^2^)	LOD (μg kg^−1^)	LOQ (μg kg^−1^)	Linear Range (ng mL^−1^)	*Correlation Coefficient* (*R*^2^)	LOD (μg kg^−1^)	LOQ (μg kg^−1^)	Linear Range (ng mL^−1^)	*Correlation Coefficient* (*R*^2^)	LOD (μg kg^−1^)	LOQ (μg kg^−1^)
AOH	0.1–200	0.996	-	-	0.2–200	0.997	0.08	0.18	0.3–200	0.994	0.12	0.29
AME	0.1–200	0.994	-	-	0.3–200	0.996	0.11	0.28	0.3–200	0.999	0.13	0.30
ALT	0.1–200	0.999	-	-	0.1–200	0.995	0.05	0.10	0.2–200	0.999	0.08	0.18
TEN	0.1–200	0.997	-	-	0.1–200	0.999	0.03	0.09	0.1–200	0.994	0.04	0.10
TeA	0.1–200	0.999	-	-	0.2–200	0.999	0.09	0.19	0.3–200	0.999	0.11	0.25
ALS	0.1–200	0.993	-	-	0.3–200	0.992	0.11	0.30	0.5–200	0.996	0.21	0.48

**Table 3:** Recoveries and precisions of the six *Alternaria* toxins in grapes (*n* = 6).

*Alternaria* Toxins	Spiked Levels (μg kg^−1^)	Green Grape			Red Grape		
		Recovery (Mean ± SD, %)	Intra-Day Precision (RSD, %)	Inter-Day Precision (RSD, %)	Recovery (Mean ± SD, %)	Intra-Day Precision (RSD, %)	Inter-Day Precision (RSD, %)
AOH	10	79.8 ± 8.1	2.5	5.1	81.4 ± 4.6	5.1	5.7
	50	100.4 ± 2.9	3.8	12.9	95.6 ± 5.7	2.8	3.4
	100	88.3 ± 4.4	11.9	3.7	88.2 ± 2.1	9.6	6.6
AME	10	92.1 ± 3.1	5.4	12.0	94.1 ± 1.9	4.9	7.4
	50	89.7 ± 4.9	2.7	5.1	94.6 ± 5.8	6.8	8.1
	100	101.6 ± 1.8	3.0	3.8	100.1 ± 3.3	5.1	3.8
ALT	10	78.4 ± 6.9	5.9	8.7	86.7 ± 5.7	3.8	10.4
	50	80.8 ± 5.4	12.2	5.4	93.4 ± 6.1	10.1	6.7
	100	82.9 ± 1.2	4.8	7.8	90.1 ± 2.1	2.8	5.5
TEN	10	92.1 ± 2.8	5.4	5.3	98.6 ± 0.9	9.9	6.9
	50	89.4 ± 4.5	4.7	6.5	94.5 ± 1.4	6.8	10.8
	100	93.0 ± 9.1	9.2	3.7	96.7 ± 2.7	2.7	5.6
TeA	10	79.5 ± 5.6	4.1	6.9	87.2 ± 7.5	1.9	3.9
	50	81.7 ± 4.9	2.9	4.1	77.8 ± 3.4	5.4	8.9
	100	100.8 ± 2.7	5.8	5.8	95.7 ± 5.5	3.8	4.1
ALS	10	79.2 ± 5.9	9.8	6.9	86.3 ± 1.1	11.4	2.9
	50	80.0 ± 5.1	6.4	7.1	79.8 ± 2.3	5.6	4.1
	100	81.5 ± 2.8	3.7	4.0	90.2 ± 1.9	8.7	8.1

**Table 4:** Occurrence of the six *Alternaria* toxins in various grape samples.

Grape Variety	Total Amount	AOH		AME		ALT		TEN		TeA		ALS	
		Positive	Range (μg kg^−1^)	Positive	Range (μg kg^−1^)	Positive	Range (μg kg^−1^)	Positive	Range (μg kg^−1^)	Positive	Range (μg kg^−1^)	Positive	Range (μg kg^−1^)
Kyoho	6	0	nd ^a^	0	nd	0	nd	0	nd	1	0.76	0	nd
Summer Black	16	2	0.12~7.15	0	nd	4	0.10~0.32	10	0.10~1.64	7	0.32~46.97	1	0.22
Shenhua	5	2	0.09–0.12	0	nd	0	nd	3	0.29~0.59	0	nd	0	nd
Hupei No.1	3	1	0.80	0	nd	0	nd	1	0.15	1	1.15	0	nd
Shenfeng	2	1	0.13	0	nd	0	nd	2	0.44~1.39	0	nd	0	nd
Muscat Hamburg	4	2	0.13~0.37	0	nd	0	nd	0	nd	1	0.60	0	nd
Shenyu	5	1	0.09	0	nd	0	nd	1	0.29	1	0.35	1	0.21
Zuijinxiang	10	4	0.11~0.28	2	0.11~0.15	1	0.53	2	0.28~0.55	3	0.25~4.39	1	0.42
Gold Finger	5	2	0.23~0.44	0	nd	1	0.18	2	0.17~0.31	2	0.38~4.61	0	nd

^a^ nd = not detected.

## Supplementary Materials

The following are available online at http://www.mdpi.com/2072-6651/11/2/87/s1, Figure S1: MS/MS spectra for AOH with the collision energy of 28 eV (A), for AME with the collision energy of 26 eV (B), for ALT with the collision energy of 14 eV (C), for ALS with the collision energy of −20 eV (D), for TEN with the collision energy of 12 eV (E) and for TeA with the collision energy of 16 eV (F). The concentration for all *Alternaria* toxins was 200 ng mL^−1^. Figure S2: Recoveries of the six *Alternaria* toxins in a standard solution filtered by different membrane filters. The concentration is 50 μg kg^−1^ (*n* = 6). Figure S3: Chemical structures of the six *Alternaria* toxins.
