# Supplementary material for: Development of a QuEChERS-Based UHPLC-MS/MS Method for Simultaneous Determination of Six Alternaria Toxins in Grapes
Source: Toxins (Basel). 2019 Feb 1;11(2):87. doi: 10.3390/toxins11020087 (PMC6410285; doi:10.3390/toxins11020087)
Supplement: Supplementary file 1 [file toxins-11-00087-s001.pdf]

Supplementary Materials: Development of a QuEChERS-Based UHPLC-MS/MS Method for Simultaneous Determination of Six *Alternaria* Toxins in Grapes

Wenbo Guo, Kai Fan, Dongxia Nie, Jiajia Meng, Qingwen Huang Junhua Yang, Yuanyuan Shen, Emmanuel K. Tangni, Zhihui Zhao, Yongjiang Wu and Zheng Han

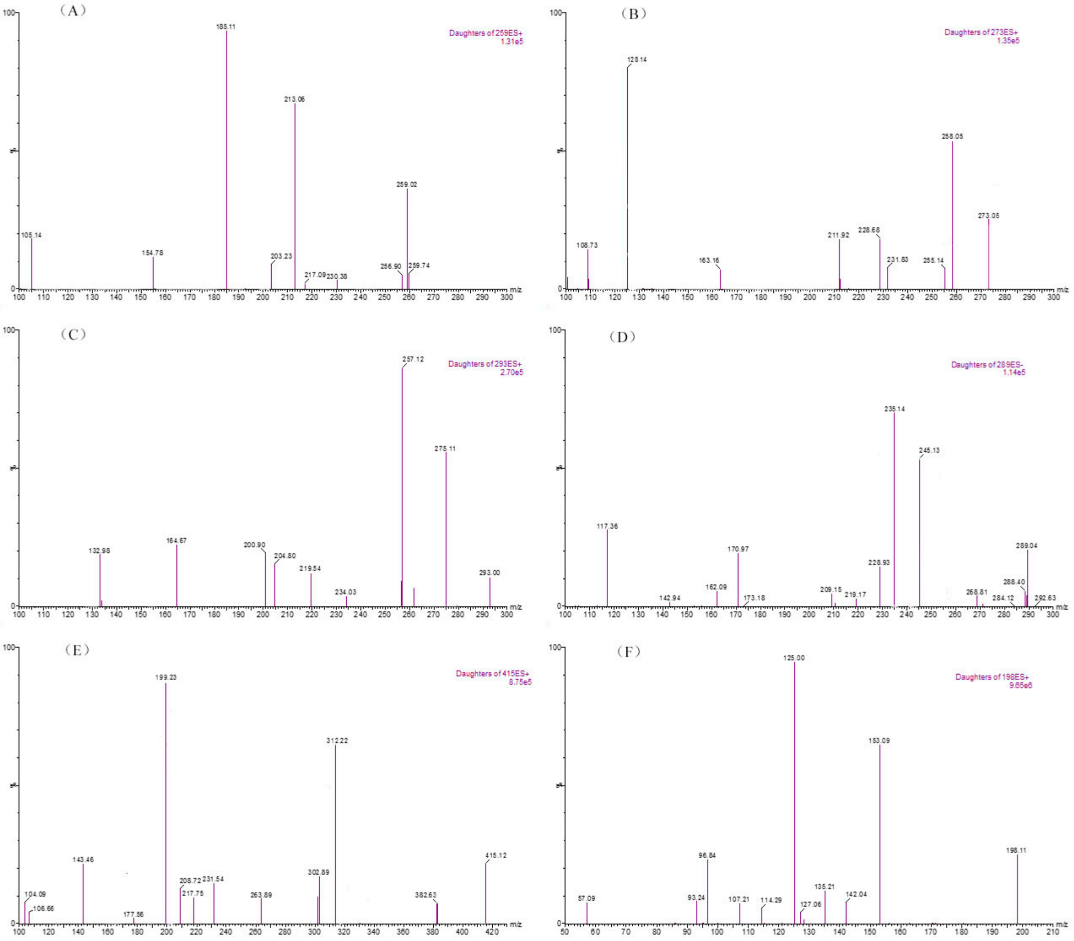

Figure 1. MS/MS spectra for AOH with the collision energy of 28 eV (**A**), for AME with the collision energy of 26 eV (**B**), for ALT with the collision energy of 14 eV (**C**), for ALS with the collision energy of −20 eV (**D**), for TEN with the collision energy of 12 eV (**E**) and for TeA with the collision energy of 16 eV (**F**). The concentration for all *Alternaria* toxins was 200 ng mL^{-1}.

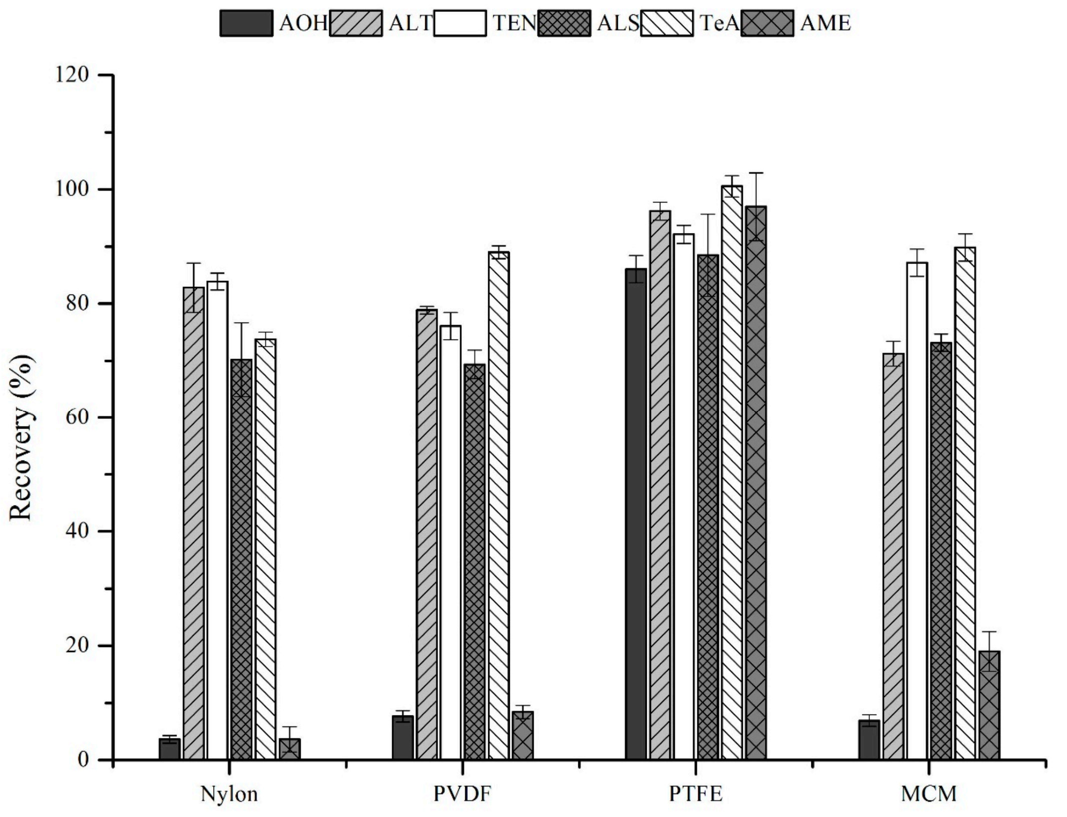

Figure 2. Recoveries of the six *Alternaria* toxins in a standard solution filtered by different membrane filters. The concentration is 50 μg kg^{-1} (n = 6).

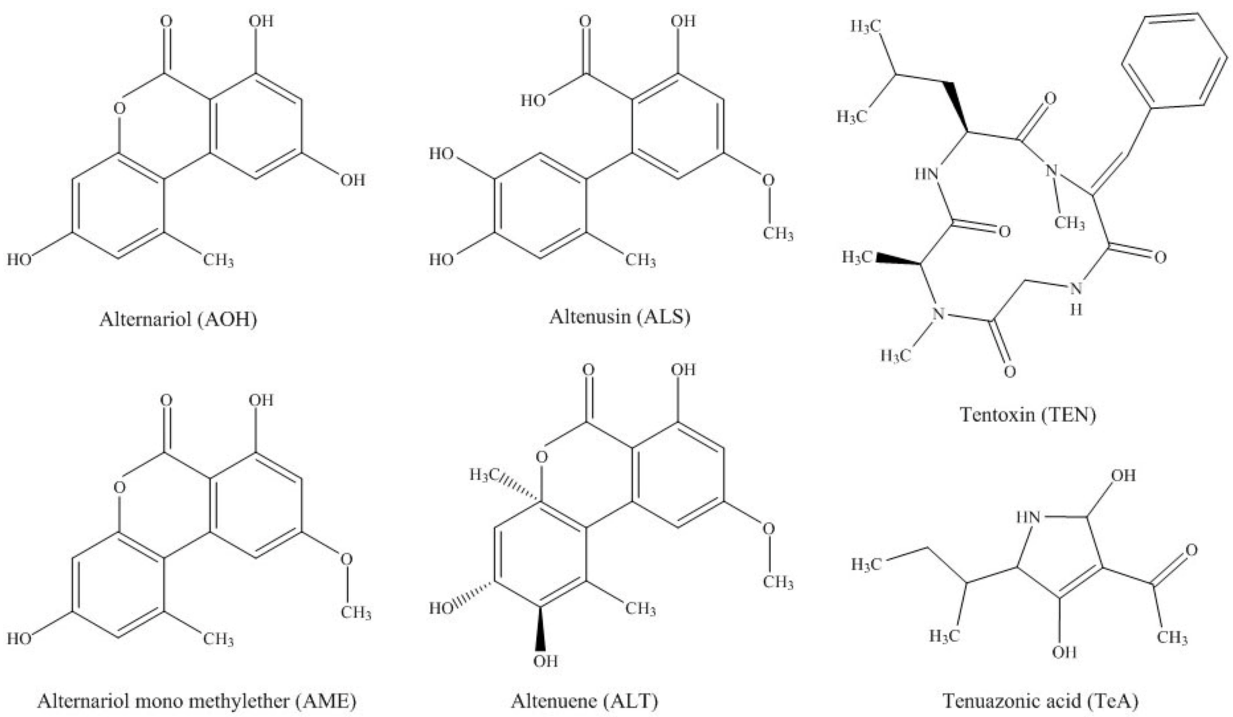

Figure 3. Chemical structures of the six *Alternaria* toxins.
